# Acute Pancreatitis in Pediatric Acute Lymphoblastic Leukemia (AcuPA Study): A Nationwide Survey in Poland

**DOI:** 10.3390/cancers16152640

**Published:** 2024-07-24

**Authors:** Anna Morawiak, Małgorzata Salamonowicz-Bodzioch, Aleksandra Królak, Krzysztof Kałwak, Joanna Owoc-Lempach, Jerzy Kowalczyk, Joanna Zawitkowska, Tomasz Szczepański, Ninela Irga-Jaworska, Elżbieta Adamkiewicz-Drożyńska, Katarzyna Albrecht, Anna Szmydki-Baran, Walentyna Balwierz, Małgorzata Czogała, Jacek Wachowiak, Katarzyna Derwich, Wojciech Młynarski, Beata Zalewska-Szewczyk, Maryna Krawczuk-Rybak, Małgorzata Sawicka-Żukowska, Jan Styczyński, Andrzej Kołtan, Krzysztof Safranow, Tomasz Urasiński, Tomasz Ociepa

**Affiliations:** 1 Department of Pediatrics, Hemato-Oncology and Gastroenterology, Pomeranian Medical University, Unii Lubelskiej 1, 71-252 Szczecin, Poland; a_morawiak@onet.eu (A.M.); aleksandra.krolak@pum.edu.pl (A.K.); tomasz.urasinski@pum.edu.pl (T.U.); 2 Department of Pediatric, Hematology, Oncology and BMT, Wrocław Medical University, 50-367 Wroclaw, Poland; msalamoniwicz@poczta.onet.pl (M.S.-B.); krzysztof.kalwak@umw.edu.pl (K.K.); joanna.owoc-lempach@umed.wroc.pl (J.O.-L.); 3 Department of Pediatric Hematology, Oncology and Transplantology, Medical University of Lublin, 20-059 Lublin, Poland; jerzy.kowalczyk@uszd.lublin.pl (J.K.); joanna.zawitkowska@umlub.pl (J.Z.); 4 Department of Pediatric Hematology and Oncology, Silesian Medical University, 40-055 Zabrze, Poland; szczep57@poczta.onet.pl; 5 Department of Pediatrics, Hematology and Oncology, Medical University, 80-210 Gdansk, Poland; ninela.irga-jaworska@gumed.edu.pl (N.I.-J.); elzbieta.adamkiewicz-drozynska@gumed.edu.pl (E.A.-D.); 6 Department of Pediatric Hematology and Oncology, Medical University, 02-091 Warszawa, Poland; katarzyna.albrecht@uckwum.pl (K.A.); anna.szmydki@uckwum.pl (A.S.-B.); 7 Department of Pediatric Oncology and Hematology, University Children’s Hospital, Collegium Medicum Jagiellonian University, 31-008 Krakow, Poland; balwierz@mp.pl (W.B.); malgorzata.czogala@uj.edu.pl (M.C.); 8 Department of Pediatric Oncology, Hematology and Transplantology, University of Medical Sciences, 61-701 Poznan, Poland; jacek.wachowiak@ump.edu.pl (J.W.); katarzyna.derwich@onkologia-dziecieca.pl (K.D.); 9 Department of Pediatrics, Hematology and Oncology, Medical University, 90-419 Lodz, Poland; wojciech.mlynarski@umed.lodz.pl (W.M.); beata.zalewska-szewczyk@umed.lodz.pl (B.Z.-S.); 10 Department of Pediatrics, Oncology an Hematology, Medical University, 15-089 Bialystok, Poland; rybak@umb.edu.pl (M.K.-R.); mzukowska@interia.pl (M.S.-Ż.); 11 Department of Pediatrics, Hematology and Oncology, Collegium Medicum in Bydgoszcz, Nicolaus Copernicus University in Torun, 87-100 Toruń, Poland; jstyczynski@cm.umk.pl (J.S.); a.koltan@cm.umk.pl (A.K.); 12 Biostatistics Teaching Unit, Pomeranian Medical University, 70-204 Szczecin, Poland; krzysztof.safranow@pum.edu.pl

**Keywords:** acute lymphoblastic leukemia, children, acute pancreatitis, risk factors, intensive chemotherapy, asparaginase

## Abstract

**Simple Summary:**

Acute lymphoblastic leukemia (ALL) is the most common childhood malignancy. Survival rates for children with ALL have improved, and today, more than 90% of children with ALL can be successfully treated. Acute pancreatitis (AP), which develops during chemotherapy for ALL can be a serious and fatal complication. It is a widely known toxicity, and several reports indicate that AP occurs with an incidence of 2–10% in patients treated for leukemia. It is mainly, but not exclusively, associated with the administration of asparaginase (ASP), which is one of the key components of multiagent chemotherapy used in ALL. The development of AP usually delays subsequent courses of chemotherapy and is the most common reason for the discontinuation of ASP, thus increasing the potential risk of leukemia relapse in children with ALL. Therefore, both the pathogenesis of AP in ALL and its impact on long-term outcomes remain to be thoroughly investigated.

**Abstract:**

Purpose: This study aimed to identify the risk factors for acute pancreatitis (AP) and its impact on outcomes in Polish children treated for ALL. Methods: The study group included 2303 children receiving intensive chemotherapy for ALL. The group was divided into patients with at least one episode of AP and those who did not develop AP after treatment for ALL. Results: The cumulative incidence of AP in the study group was 4.08%. Older age was an independent risk factor for the development of AP (OR = 1.05; 95%CI = 1.006–1.098; *p* = 0.03). The overall mortality associated with AP was 2.13%. The probabilities of disease-free survival (p-DFS) and event-free survival (p-EFS) in both subgroups were 0.84 vs. 0.86, log-rank *p* = 0.65 and 0.75 vs. 0.80, log-rank *p* = 0.12, respectively. A total of 22 out of 94 patients (23.4%) with AP were re-exposed to asparaginase (ASP) during the subsequent treatment phases. Only one patient re-exposed to ASP (4.5%) developed a second episode of AP. There were no significant differences in p-DFS and p-EFS between patients re-exposed and not re-exposed to asparaginase (0.78 vs. 0.86, log-rank *p* = 0.27 and 0.63 vs. 0.79, log-rank *p* = 0.09, respectively). Conclusions: The incidence of AP in children with ALL is low and related to patients’ age. The development of AP does not seem to influence p-DFS and p-EFS in children with ALL. Recurrence of AP after re-exposure to asparaginase in patients with ALL and a history of AP is low (4.5%). Re-exposure to asparaginase after the first episode of AP does not improve either p-DFS or p-EFS in children with ALL.

## 1. Introduction

Acute lymphoblastic leukemia (ALL) is the most common malignancy in children [1]. The introduction of intensive chemotherapy, better risk stratification, and improved supportive care have improved the survival of children with ALL, and today, more than 90% children with ALL can be successfully treated [2,3,4,5]. However, more intensive treatment is associated with an increased prevalence of toxicities, which have been shown to be a significant cause of non-cancer morbidity and mortality in this patient population [6,7].

Acute pancreatitis (AP) developing during chemotherapy for ALL can be a serious and fatal complication. It is a widely recognized toxicity, and several reports indicate that AP occurs with an incidence of 2–10% in patients treated for leukemia [8,9]. It is mainly, but not exclusively, associated with the administration of asparaginase, which is one of the key components of multiagent chemotherapy used in ALL [9,10]. AP development delays subsequent courses of chemotherapy and is the most common reason for the discontinuation of ASP, thus increasing the potential risk of leukemia relapse in children with ALL [9,11,12,13]. Parenchymal toxicity of chemotherapy has been suggested to be a causative factor in the development of AP [14,15]. Older age, the type of asparaginase used, and higher leukemia risk stratification are indicated as potential risk factors for the development of acute pancreatitis [8,9,10,12]. However, the pathogenesis of AP in ALL and its impact on long-term outcomes remain to be thoroughly investigated. Furthermore, the prediction of AP in children with ALL is not feasible in routine clinical practice [6,8,10,14,15].

The aim of this study was to identify the risk factors for acute pancreatitis, its characteristics, and impact on outcomes in Polish children treated for ALL using two consecutive protocols, ALL IC BFM 2002 and 2009.

## 2. Materials and Methods

### 2.1. Patients’ Selection

Between 2005 and 2015, 2303 children (1003 girls and 1300 boys, aged 1–18 years; median age = 5.1) were diagnosed with de novo ALL and received intensive chemotherapy according to ALL IC BFM 2002 and 2009 protocols in all 16 centers of the Polish Pediatric Leukemia/Lymphoma Study Group (PPLLSG). All these children were included in the study group. Treatment according to ALL IC BFM 2002 and ALL IC BFM 2009 protocols for standard-, intermediate-, and high-risk groups is summarized in Supplementary Tables S1 and S2. A native formulation of *E. coli* l-asparaginase was used as frontline therapy in both protocols. The dose and frequency of ASP administration varied according to the risk group stratification. The most intensive chemotherapy was given to the patients in the high-risk group. Features that were still used in the high-risk stratification were poor prednisone response (PPR), ≥5% bone marrow blasts on day 33, flow cytometric minimal residual disease detection (FC-MRD) on day 15 > 10% (only for ALL IC BFM 2009 protocol), and the presence of t(4;11) or t(9;22). In the case of hypersensitivity to ASP, the drug was replaced by Erwinia chrysanthemi asparaginase.

The Atlanta criteria, modified for children by the INSPPIRE group, were used to diagnose AP [16,17]. AP was diagnosed when at least two of three of the following criteria were met:

1. Abdominal symptoms suggestive of acute pancreatitis;
2. Serum amylase or lipase levels three times above the upper limit of normal levels;
3. Imaging findings: ultrasonography (USG), computer tomography (CT), or magnetic resonance imaging (MRI) indicating acute pancreatitis.

The study group was subdivided into patients with at least one episode of acute pancreatitis during antileukemic treatment (Group 1) and those who did not develop AP after treatment for leukemia (Group 2).

### 2.2. Statistical Analysis

Data were expressed and described as absolute numbers, percentages, and mean ± SD. The continuous variables were compared between groups using the two-tailed Mann–Whitney test. The logistic regression model was used to identify risk factors for the development of acute pancreatitis, and multivariate regression analysis was performed to identify multiple independent risk factors. Fisher’s exact test was used to compare unpaired, nominal variables. Kaplan–Meier method and the log-rank test were used for survival analysis. *p* values ≤ 0.05 were considered significant. Statistical analyses were performed using the Statistica software (TIBCO Software Inc., Stanford Research Park, Palo Alto, CA, USA, ver. 13).

## 3. Results

Acute pancreatitis was diagnosed in 94 of 2303 patients (43 girls and 51 boys, aged 1–18 years; median age = 7.2) treated for ALL. The cumulative incidence of AP development was 4.08% (95%CI = 3.31–4.97), and the annual incidence of AP was 371/100,000 children with ALL. The majority of acute pancreatitis episodes (62 out of 94, 66%) occurred during induction protocol I. Only 6 (6.4%) AP episodes developed during protocol M, 13 (13.8%) during protocol II, 12 (12.8%) during HR 1–3 protocols, and 1 (1%) during maintenance treatment. Overall, 87 out of 94 episodes of acute pancreatitis (92.6%) were associated with ASP therapy during protocols I and II and intensive protocols for the high-risk group. The median time for the development of AP was 37 days (range: 6–778 days, mean: 102 days) from the start of treatment. The median number of ASP doses administered before AP development was 8 (range: 1–15; mean: 7.75 ± 3.53). Most APs occurred during induction protocol I, and the median number of ASP doses administered before AP development was 6.5 (range: 1–8; mean: 5.9 ± 2.34). Treatment of acute pancreatitis in the study group was primarily supportive, with 80.9% of patients (76/94) receiving antibiotics, 76.6% of patients (72/94) receiving parenteral nutrition, and 14.9% (14/94) receiving a somatostatin analog (octreotide). The choice of treatment was at the discretion of the treating physician.

Group 1 and Group 2 were not statistically different with respect to gender (*p* = 0.74), therapeutic group distribution/ALL risk stratification (*p* = 0.13), and treatment protocol (*p* = 0.60). Univariate analysis showed that patients who developed AP during ALL treatment were significantly older at the time of ALL diagnosis (8.0 ± 4.6 vs. 6.7 ± 4.6 years; *p* = 0.004) and had T-lineage ALL (19.1% vs. 11.8%; *p* < 0.01). These data are shown in Table 1.

In the logistic regression model, older age but not T-lineage phenotype retained an independent influence on the development of AP (OR = 1.05; 95%CI = 1.006–10.98; *p* = 0.03 and OR = 1.631; 95%CI = 0.937–2.839; *p* = 0.08, respectively). The multivariable regression model showed that age was associated with the development of AP, with each year increasing the risk of acute pancreatitis by 5.1%, and that the incidence of AP was significantly higher in children 7 years and older compared to younger children (6.06% vs. 2.7%; *p* < 0.01; OR = 0.437; 95%CI = 0.2873–0.664). These data are shown in Table 2.

**Figure 1:**
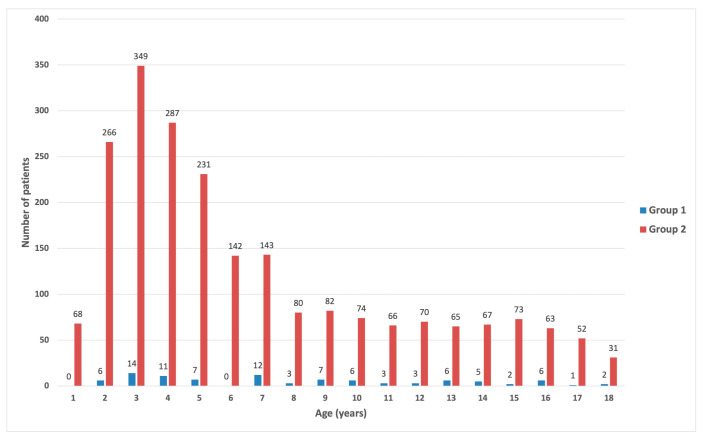
The distribution of acute pancreatitis across the age groups.

The distribution of acute pancreatitis across the age groups in the study cohort is presented in Figure 1.

A total of 51 acute and chronic complications of AP were recorded in 29 (30.9%) patients with AP. The most common complications were diabetes (10/94; 10.6%), pancreatic pseudocyst (9/94; 9.6%), acute peritonitis (7/94; 7.5%), hemorrhagic necrosis (6/94; 6.4%), pleural effusion (5/94; 5.3%), multiorgan failure (3/94; 3.25%), and ileus (2/94; 2.1%). Only 2 of 94 patients with AP died from multiorgan failure directly caused by acute pancreatitis. These data are shown in Table 3.

**Figure 2:**
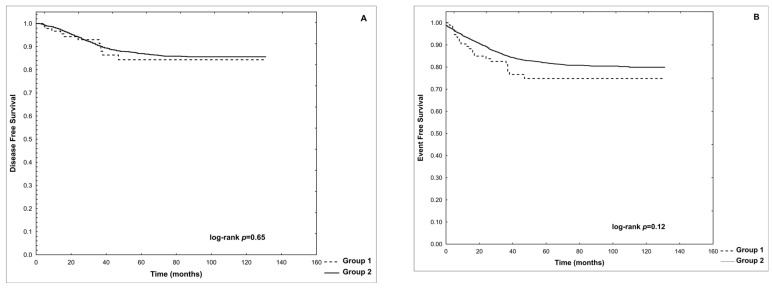
Probability of disease-free survival (**A**) and event-free survival (**B**) in Groups 1 and 2.

The risk of death in Group 1 was higher than in Group 2 (17/94 vs. 232/2209; RR = 1.7220; 95%CI = 1.1011–2.6929; *p* = 0.02). The overall mortality related to AP in Group 1 was 2.13%; it accounted for 2/17 (11.7%) of all deaths in Group 1. The overall mortality related to AP in the entire cohort of 2303 patients was 0.087%. The probabilities of disease-free survival (p-DFS) and event-free survival (p-EFS) in Group 1 were not significantly different from those in Group 2 (0.84 vs. 0.86, log-rank *p* = 0.65 and 0.75 vs. 0.80, log-rank *p* = 0.12, respectively). These data are shown in Figure 2.

A total of 22 of 94 patients (23.4%) with AP after recovery were re-exposed to ASP during the subsequent phases of the treatment. All re-exposed patients received the exact formulation of asparaginase as used in previous treatment phases. There were no significant differences between patients re-exposed (ASP+) or not re-exposed (ASP−) to ASP in age at the time of ALL diagnosis (7.41 ± 4.07 vs. 8.08 ± 4.80; *p* = 0.55), male gender (59.1% vs. 52.1%; *p* = 0.63), and T-lineage ALL phenotype (18.2% vs. 19.7%; *p* = 0.84). The number of patients with the mild course of the first episode of acute pancreatitis was similar in both groups (15/22 vs. 49/72; *p* = 1.00). These data are shown in Table 4.

**Figure 3:**
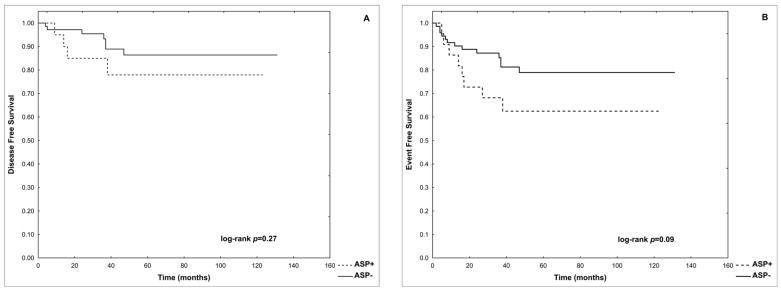
The probability of disease-free survival (**A**) and event-free survival (**B**) in ASP+ and ASP− groups.

Only 1 of 22 patients re-exposed to ASP (4.5%) developed a second episode of AP. There were no significant differences in p-DFS and p-EFS between patients re-exposed (ASP+) and not re-exposed to asparaginase (ASP−) after the first episode of AP (0.78 vs. 0.86, log-rank *p* = 0.27 and 0.63 vs. 0.79, log-rank *p* = 0.09, respectively). These data are shown in Figure 3.

## 4. Discussion

Acute pancreatitis is a serious and relatively common complication of anti-leukemic therapy in children [8,9,13,18]. It is mostly, but not always, associated with the administration of asparaginase, an essential component of intensive therapy for acute lymphoblastic leukemia [9,11,13,15,18]. The incidence of AP after ALL treatment reported in several studies varies from 1.5% to as high as 18% [14,18,19,20,21]. In the present study, the overall incidence of AP in children treated for ALL was 4.08%. In 92.6% of cases, pancreatitis was associated with ASP therapy during protocols I and II and intensive protocols for the high-risk group. Thus, the overall incidence of asparaginase-associated pancreatitis in children treated for ALL was 3.7%. This incidence contrasts with that of the study of Rank et al. which found that the cumulative incidence of first-time asparaginase-associated AP in patients with ALL was 8.3% (95%CI = 7.0 to 9.9). This is almost 2.3 times higher than in our study population [19]. Such a relatively high incidence of AP reported by Rank et al. may be explained by the fact that Rank’s study group included not only children but also adults. However, it is also worth mentioning that contrary results were reported by Liu et al. and Samarasinghe et al. They also studied a cohort of children and young adults with ALL and found that the risk of developing AP was only 2.3% and 1.5%, respectively [20,21]. The relatively low incidence of AP in our study group may be explained by the fact that l-asparaginase was used as a standard formulation in the study. Alvarez et al. confirmed that the use of pegylated asparaginase (PEG-ASP), the drug with a longer half-life, is associated with a significant increase in the incidence of pancreatitis compared to the classical formulation of l-asparaginase (18% vs. 1.9%), probably due to prolonged asparagine depletion [11].

The time to AP development was also analyzed in our study. We found that the median time to AP development was 37 days, meaning that at least 50% of AP episodes occurred after eight doses of ASP scheduled for the induction phase (Supplementary Table S1). We also found that the median number of ASP doses given before AP development was 8 (range: 1–15; mean: 7.75). It is worth mentioning that most AP episodes occurred during induction protocol I, with the median number of ASP doses given prior to AP development being 6.5 (range 1–8; mean: 5.9).

Analysis of several potential risk factors for the development of AP, including gender, therapeutic group, treatment protocol, and leukemia immunophenotype, showed that older age at the time of ALL diagnosis was the only independent risk factor for the development of acute pancreatitis in children with ALL, and that the rate of AP was significantly higher in children older than six years as compared to younger children. This is partially consistent with data published by Kearney et al. They confirmed that children with ALL older than ten years have an increased risk of developing AP compared to younger children [22]. Similar observations have been reported by others [12,21]. However, it is also worth mentioning that Raja et al. found that the development of AP in children with ALL in the NOPHO ALL2008 protocol was not significantly associated with the patient’s age [9].

Several studies confirmed the association of AP with ALL risk stratification. Samarasinghe et al. found that patients with high-risk ALL had a higher frequency of AP than standard-risk patients. The authors speculated that this was related to the cumulative dose of ASP, since patients with high-risk ALL received a higher cumulative dose of ASP [21]. Liu et al. also confirmed that a higher cumulative dose of ASP was an independent risk factor for the development of AP [20]. This is consistent with the observation of Raja et al. In this study, the high-risk ALL patients treated according to the NOPHO ALL2008 protocol received a lower cumulative dose of ASP. The rate of AP was also lower in this group of patients [9]. On the contrary, our results did not confirm a higher frequency of AP in high-risk ALL, although patients stratified into a high-risk group received a higher cumulative dose of ASP in ALL IC BFM 2002 and 2009 protocols [Supplementary Tables S1 and S2].

Because the use of asparaginase in the treatment of ALL improves event-free survival, its early discontinuation due to AP or other toxicities may be associated with worse outcomes [23,24,25]. Although asparaginase was discontinued in 76.6% of patients with AP in our study, both disease- and event-free survivals were comparable to patients who received remaining doses of ASP in the subsequent phases of therapy. This may be explained by the fact that at least 50% of all patients with AP received all eight doses of ASP scheduled for induction. The remaining chemotherapeutic agents are likely to be effective enough to achieve and maintain complete remission. This may be consistent with data published by Dos Santos et al. They found out that children with ALL who received less than ten doses of ASP may be at an increased risk of treatment failure only if they were in a high-risk group. However, this study did not show statistical significance in the number of asparaginase doses in children with ALL who were standard- or intermediate-risk [26]. The above mentioned, as well as our results, may indicate that the occurrence of acute pancreatitis in children with ALL is not a risk factor for leukemia relapse.

Acute pancreatitis in children may lead to severe complications, including systemic inflammatory response syndrome, multiorgan failure, shock, acute respiratory distress syndrome, severe sepsis, pancreatic necrosis, renal failure, and others [9,11]. Immunosuppressed children with cancer are thought to be at risk of a severe course of acute pancreatitis with high mortality rate. The treatment of acute pancreatitis in children with ALL is primarily supportive and consists of early and adequate fluid resuscitation, pain relief, and proper nutrition [8,13,15,21,22,26]. If sepsis is suspected, broad-spectrum antibiotics are recommended [8]. Notably, some studies and case series reports have shown a promising role for a somatostatin analog (octreotide) in the treatment and prevention of asparaginase-associated AP, offering a potential opportunity for improved outcomes [26,27,28]. Our study was not designed to analyze the response of patients to treatment for acute pancreatitis. However, it needs to be specified that 14 of 94 patients with AP (14.9%) received octreotide.

Although there was relatively significant morbidity in our cohort (10.6% developed diabetes mellitus, 9.6% developed pseudocyst, 5.3% pleural effusion, and 3.2% multiorgan failure), the overall mortality related to AP was 2.13% for the group of patients with AP and 0.089% for the entire cohort of children with ALL. We found that the risk of death was higher in patients with AP compared to the rest of the cohort and that AP accounted for 11.7% of all deaths, making AP a significant cause of death in children with ALL complicated by acute pancreatitis. It is at least partially in line with data published by Rank et al. [19]. However, it should also be noted that AP-related mortality reported by Wang et al. was almost three times higher (5.71%) compared to our cohort [18].

One-quarter of the patients with AP (22/94; 23.4%) were re-exposed to ASP after recovery during subsequent treatment phases, of which only one (1/22; 4.5%) developed a second episode of AP. These data contrast with observations of Raja et al., who found a 17% risk of a second episode of AP in patients rechallenged with ASP [9]. A higher incidence of AP after re-exposure to ASP was also reported by Rank et al. [19]. They found that up to 44% of rechallenged ASP patients developed a second episode of AP. The higher rate of a second episode of AP in the study of Rank et al. may be partially explained by the fact that they used PEG-ASP formulation in the study [19]. Reintroduction of ASP after resolution of AP in children with ALL was also studied by Kearney et al. [22]. They found that 63% of children re-exposed to ASP developed a second episode of AP. It is worth mentioning that, in all these studies, the course of a second AP was mild in most of the patients [9,19,22]. Although ASP was withdrawn in 76.6% of patients after the first episode of AP, there were no significant differences in disease-free and event-free survival in these patients compared to those re-exposed to ASP. This is consistent with the results of Samarasinghe et al. who found that ASP withdrawal due to AP did not affect event-free and overall survival in children and young adults with ALL [21]. It should also be emphasized that it contrasts with data published by Silverman et al., who found a lower event-free survival in pediatric ALL after the discontinuation of ASP due to its toxicity [24]. Considering all these data, the question of whether the discontinuation of ASP after the first episode of AP may influence event-free or disease-free survival remains unanswered.

The main strength of this study is its multicenter design, which included 2303 uniformly treated consecutively diagnosed children with acute lymphoblastic leukemia. To our knowledge, this is one of the largest studies that includes such a large group of children with ALL. This leads us to believe that the results are reliable.

Limitations include the retrospective study design and the sample size of re-exposed ASP patients, which limits definitive conclusions regarding disease-free and event-free survival in this group of ALL patients. Due to the small sample size (only one patient developed a second episode of AP), it was impossible to analyze potential risk factors for a second AP development.

## 5. Conclusions

In summary, we have shown that the incidence of acute pancreatitis in children treated for acute lymphoblastic leukemia is relatively low and related to the patient’s age.

We have also shown that the development of AP during chemotherapy and the truncation of AP does not affect disease-free and event-free survival.

We also found that the recurrence of AP in patients re-exposed to ASP after the first episode of AP is low.

## Figures and Tables

**Table 1:** Characteristics of the study groups.

		Group 1 (*n* = 94)	Group 2 (*n* = 2209)	*p* Value
Age (years) mean ± SD [median]	at the time of ALL diagnosis	8.0 ± 4.6 [7.2]	6.7 ± 4.5 [5.0]	<0.01
	at the time of AP diagnosis	8.1 ± 4.7 [7.2]	NA	NA
Gender (no. of patients)	male	51 (54.3%)	1249 (56.5%)	0.74
	female	43 (45.7%)	960 (43.5%)	
ALL type (no. of patients)	B-cell	75 (79.8%)	1861 (84.3%)	<0.01
	T-cell	18 (19.2%)	250 (11.3%)	
	bilinear	1 (1.1%)	2 (0.1%)	
	no data	0	96 (4.3%)	
Therapeutic group (no. of patients)	HR	26 (27.7%)	434 (20.0%)	0.13
	IR	48 (51.1%)	1133 (52.2%)	
	SR	20 (21.3%)	604 (27.8%)	
	no data	0	38	
Treatment protocol	ALLIC 2002	60 (63.8%)	1480 (67.0%)	0.60
	ALLIC 2009	34 (36.2%)	729 (33.0%)	
Stage of treatment at which first AP was diagnosed	Protocol I	62 (66.0%)	NA	NA
	Protocol M	6 (6.4%)	NA	NA
	Protocol II	13 (13.8%)	NA	NA
	Protocols HR1-HR3	12 (12.8%)	NA	NA
	maintenance therapy	1 (1.0%)	NA	NA
The total number of ASP doses before AP development	Mean Median Range	7.75 ± 3.53 8 1–15	NA NA NA	NA NA NA
The total number of ASP doses before AP development during induction protocol I	Mean Median Range	5.9 ± 2.34 6.5 1–8	NA NA NA	NA NA NA
Deaths		17 (18%)	232 (11%)	0.02

SR—standard risk; IR—intermittent risk; HR—high risk; ASP—asparaginase; AP—acute pancreatitis; NA—not applicable.

**Table 2:** The association between patient’s sex, age, leukemia phenotype, treatment protocol, and AP development in logistic regression analysis.

	Male	Older Age at the Time of ALL Diagnosis	ALL-T	ALL IC-BFM 2009 Treatment Protocol
*p*	0.356	0.025	0.084	0.580
odds ratio (OR)	0.818	1.051	1.631	1.131
confidence interval (95%CI)	0.534–1.253	1.006–1.098	0.937–2.839	0.731–1.751

**Table 3:** Acute and chronic complications of AP in study group.

Complications	No. of Events (Rate)
diabetes	10 (10.6%)
pseudocysts	9 (9.6%)
peritonitis	7 (7.5%)
hemorrhagic necrosis	6 (6.4%)
pleuritis	5 (5.3%)
multiorgan failure	3 (3.2%)
gastrointestinal obstruction	2 (2.1%)
death due to AP	2 (2.1%)
other	7 (7.5%)

**Table 4:** Characteristics of patients re-exposed (ASP+) and not re-exposed (ASP−) to asparaginase after the first episode of acute pancreatitis.

		ASP+ (*n* = 22)	ASP− (*n* = 72)	*p* Value
Age mean ± SD (years) [median]	at the time of ALL diagnosis	7.41 ± 4.07 [7.1]	8.17 ± 4.14 [7.3]	0.55
Gender (no. of patients)	male	13 (59.1%)	38 (52.8%)	0.63
	female	9 (40.9%)	34 (47.2%)	
ALL type (no. of patients)	B-cell	18 (81.8%)	57 (79.2%)	0.85
	T-cell	4 (18.2%)	14 (19.4%)	
	bilinear cell	0 (0.0%)	1 (1.4%)	
Therapeutic group (no. of patients)	HR	6 (27.3%)	20 (27.8%)	0.56
	IR	13 (59.1%)	35 (48.6%)	
	SR	3 (13.6%)	17 (23.6%)	
Treatment protocol	ALLIC 2002	13 (59.1%)	47 (65.3%)	0.62
	ALLIC 2009	9 (40.9%)	25 (34.7%)	
Stage of treatment at which first AP was diagnosed	I	12 (54.5%)	50 (69.4%)	0.43
	M	1 (4.5%)	4 (5.6%)	0.61
	II	3 (13.6%)	10 (13.9%)	1.0
	HR	5 (22.7%)	7 (9.7%)	0.29
	maintenance therapy	0 (0.0%)	1 (1.4%)	1.0
	no data	1 (4.5%)	0	NA
Patients with the mild course of AP		15 (68.2%)	49 (68.1%)	1.0

NA—not applicable.

## Data Availability

Data available upon request.

## Supplementary Materials

The following supporting information can be downloaded at: https://www.mdpi.com/article/10.3390/cancers16152640/s1, Supplementary Table S1: Treatment for standard-, intermediate-, and high-risk groups according to ALL IC BFM 2002 protocol.; Supplementary Table S2: Treatment for standard-, intermediate-, and high-risk groups according to ALL IC BFM 2009 protocol.
