# Supplementary material for: Acute Pancreatitis in Pediatric Acute Lymphoblastic Leukemia (AcuPA Study): A Nationwide Survey in Poland
Source: Cancers (Basel). 2024 Jul 24;16(15):2640. doi: 10.3390/cancers16152640 (PMC11312082; doi:10.3390/cancers16152640)
Supplement: Supplementary file 1 [file cancers-16-02640-s001.zip › cancers-3044988-supplementary.pdf]

cancers MDPI

Table S1. Treatment for standard-, intermediate- and high-risk groups according to ALL IC BFM 2002 protocol.

ALL IC 2002 BFM	standard-risk group	intermediate-risk group	high-risk group
**induction therapy**	Protocol I<br>prednisone/prednisolone 60 mg/m2/d (days 1-28),<br>vincristine 1.5 mg/m2, max. 2 mg (days: 8, 15, 22, 29),<br>daunorubicin 30 mg/m2 (days 8, 15, 22, 29 – SRG ALL-T and IRG or days 8, 15 – SRG ALL-BCP)<br>E. coli, l-asparaginase 5,000 IU/m2/d (days 12, 15, 18, 21, 24, 27, 30, 33),<br>cyclophosphamide 1,000 mg/m2/d (days 36, 64),<br>6-mercaptopurine 60 mg/m2/d (days 36-63), cytarabine 75 mg/m2/d (days 38-41, 45-48, 52-55, 59-62),<br>intrathecal methotrexate, age-adjusted dosage, max. 12 mg/d (days 1, 12, 33, 45, 59), if CNS positive or traumatic LP additional doses on days 18, 27)	Protocol I<br>prednisone/prednisolone 60 mg/m2/d (days 1-28),<br>vincristine 1.5 mg/m2, max. 2 mg (days: 8, 15, 22, 29),<br>daunorubicin 30 mg/m2 (days 8, 15, 22, 29),<br>E. coli, l-asparaginase 5,000 IU/m2/d (days 12, 15, 18, 21, 24, 27, 30, 33),<br>cyclophosphamide 1,000 mg/m2/d (days 36, 64),<br>6-mercaptopurine 60 mg/m2/d (days 36-63), cytarabine 75 mg/m2/d (days 38-41, 45-48, 52-55, 59-62),<br>intrathecal methotrexate, age-adjusted dosage, max. 12 mg/d (days 1, 12, 33, 45, 59), if CNS positive or traumatic LP additional doses on days 18, 27)	Protocol I<br>prednisone/prednisolone 60 mg/m2/d (days 1-28),<br>vincristine 1.5 mg/m2, max. 2 mg (days 8, 15, 22, 29),<br>daunorubicin 30 mg/m2 (days 8, 15, 22, 29),<br>E. coli, l-asparaginase 5,000 IU/m2/d (days 12, 15, 18, 21, 24, 27, 30, 33),<br>cyclophosphamide 1,000 mg/m2/d (days 36, 64),<br>6-mercaptopurine 60 mg/m2/d (days 36-63), cytarabine 75 mg/m2/d (days 38-41, 45-48, 52-55, 59-62),<br>intrathecal methotrexate, age-adjusted dosage, max. 12 mg/d (days 1, 12, 33, 45, 59), if CNS positive or traumatic LP additional doses on days 18, 27)
**consolidation therapy**	Protocol M<br>6-mercaptopurine 25 mg/m2/d (days 1-56),<br>methotrexate 2,000 mg/m2 (days 8, 22, 36, 50) – SRG ALL-BCP or 5,000 mg/m2 (days 8, 22, 36, 50) – SRG ALL-T<br>intrathecal methotrexate, age-adjusted dosage, max. 12 mg/d (days 8, 22, 36, 50)	Protocol M<br>6-mercaptopurine 25 mg/m2/d (days 1-56),<br>methotrexate 2,000 mg/m2 (days 8, 22, 36, 50) – IRG ALL-BCP or 5,000 mg/m2 (days 8, 22, 36, 50) – IRG ALL-T<br>intrathecal methotrexate, age-adjusted dosage, max. 12 mg/d (days 8, 22, 36, 50)	Protocol HR1, HR2, HR3<br>dexamethasone 20 mg/m2/d (days 1-5 prot. HR1, HR2, HR3)<br>vincristine 1,5 mg/m2/d, max. 2 mg (days 1, 6 prot. HR1),<br>methotrexate 5,000 (day 1 prot. HR1, HR2),<br>cyclophosphamide 200 mg/m2/d (5 doses on days 2, 3, 4 prot. HR1),<br>cytarabine 2,000 mg/m2/d (2 doses on day 5 prot. HR1, 4 doses on days 1-2 prot. HR3),<br>l-asparaginase 25,000 IU/m2/d (days 6, 11 prot. HR1, HR2, HR3),<br>vindesine 3 mg/m2/d, max. 5 mg (days 1, 6 prot. HR2),<br>ifosfamide 800 mg/m2/d (5 doses on days 2, 3, 4 prot. HR2),<br>daunorubicin 30 mg/m2/d (day 5 prot. HR2),<br>etoposide 100 mg/m2/d (5 doses on days 3, 4, 5 prot. HR3),<br>intrathecal methotrexate+cytarabine+prednizol

			one, age-adjusted dosage, max. 12+30+10 mg/d (day 1 prot. HR1, HR2, HR3)
**reinduction therapy**	Protocol II<br>dexamethasone 10 mg/m2/d (days 1-21),<br>vincristine 1.5 mg/m2/d, max. 2 mg (days 8, 15, 22, 29),<br>doxorubicin 30 g/m2/d (days 8, 15, 22, 29),<br>E. coli L-asparaginase 10,000 IU/m2/d (days 8, 11, 15, 18),<br>cyclophosphamide 1,000 mg/m2/d (day 36),<br>6-thioguanine 60 mg/m2/d (days 36-49),<br>cytarabine 75 mg/m2/d (days 38-41, 45-48),<br>intrathecal methotrexate, age-adjusted dosage, max. 12 mg/d (days 38, 45), if CNS positive or traumatic LP additional doses on days 1, 18)	Protocol II<br>dexamethasone 10 mg/m2/d (days 1-21),<br>vincristine 1.5 mg/m2/d, max. 2 mg (days 8, 15, 22, 29),<br>doxorubicin 30 g/m2/d (days 8, 15, 22, 29),<br>E. coli L-asparaginase 10,000 IU/m2/d (days 8, 11, 15, 18),<br>cyclophosphamide 1,000 mg/m2/d (day 36),<br>6-thioguanine 60 mg/m2/d (days 36-49),<br>cytarabine 75 mg/m2/d (days 38-41, 45-48),<br>intrathecal methotrexate, age-adjusted dosage, max. 12 mg/d (days 38, 45), if CNS positive or traumatic LP additional doses on days 1, 18)	Protocol II<br>dexamethasone 10 mg/m2/d (days 1-21),<br>vincristine 1.5 mg/m2/d, max. 2 mg (days 8, 15, 22, 29),<br>doxorubicin 30 g/m2/d (days 8, 15, 22, 29),<br>E. coli L-asparaginase 10,000 IU/m2/d (days 8, 11, 15, 18),<br>cyclophosphamide 1,000 mg/m2/d (day 36),<br>6-thioguanine 60 mg/m2/d (days 36-49),<br>cytarabine 75 mg/m2/d (days 38-41, 45-48),<br>intrathecal methotrexate, age-adjusted dosage, max. 12 mg/d (days 38, 45), if CNS positive or traumatic LP additional doses on days 1, 18)<br><br>Interim Maintenance - 4 weeks<br>methotrexate 20 mg/m2 (once per week),<br>6-mercaptopurine 50 mg/m2/d (once per day),<br><br>Protocol II' as protocol II
maintenance therapy	methotrexate 20 mg/m2 (once per week),<br>6-mercaptopurine 50 mg/m2/d (once per day),<br>intrathecal methotrexate, age-adjusted dosage, max. 12 mg/d (every 4 weeks, 2-4 doses in selected groups of patients); no MTX i.th. if CRT indicated	methotrexate 20 mg/m2 (once per week),<br>6-mercaptopurine 50 mg/m2/d (once per day),<br>intrathecal methotrexate, age-adjusted dosage, max. 12 mg/d (every 4 weeks, 2-4 doses in selected groups of patients) ; no MTX i.th. if CRT indicated	methotrexate 20 mg/m2 (once per week),<br>6-mercaptopurine 50 mg/m2/d (once per day),<br>therapeutic or prophylactic CRT
Cumulative asparaginase dose	80 000 u/m2	80 000 u/m2	270 000 u/m2

Table S2. Treatment for standard-, intermediate- and high-risk groups according to ALL IC BFM 2009 protocol.

ALL IC 2009 BFM	standard-risk group	intermediate-risk group	high-risk group
**induction therapy**	Protocol I<br>prednisone/prednisolone 60 mg/m2/d (days 1-28),<br>vincristine 1.5 mg/m2, max. 2 mg (days 8, 15, 22, 29),<br>daunorubicin 30 mg/m2 (days 8, 15, 22, 29 – SRG ALL-T or days 8, 15 only – SRG ALL-BCP)<br>E. coli, l-asparaginase 5,000 IU/m2/d (days 12, 15, 18, 21, 24, 27,<br>30, 33), cyclophosphamide 1,000 mg/m2/d (days 36, 64),<br>6-mercaptopurine 60 mg/m2/d (days 36-63),<br>cytarabine 75 mg/m2/d (days 38-41, 45-48, 52-55, 59-62),<br>intrathecal methotrexate, age-adjusted dosage, max. 12 mg/d (days 1, 12, 33, 45, 59, if CNS positive or traumatic LP additional doses on days 18, 27)	Protocol I<br>prednisone/prednisolone 60 mg/m2/d (days 1-28),<br>vincristine 1.5 mg/m2, max. 2 mg (days 8, 15, 22, 29),<br>daunorubicin 30 mg/m2 (days 8, 15, 22, 29),<br><br>E. coli, l-asparaginase 5,000 IU/m2/d (days 12, 15, 18, 21, 24, 27,<br>30, 33), cyclophosphamide 1,000 mg/m2/d (days 36, 64),<br>6-mercaptopurine 60 mg/m2/d (days 36-63),<br>cytarabine 75 mg/m2/d (days 38-41, 45-48, 52-55, 59-62),<br>intrathecal methotrexate, age-adjusted dosage, max. 12 mg/d (days 1, 12, 33, 45, 59, if CNS positive or traumatic LP additional doses on days 18, 27)	Protocol I<br>prednisone/prednisolone 60 mg/m2/d (days 1-28),<br>vincristine 1.5 mg/m2, max. 2 mg (days 8, 15, 22, 29, 50, 57, 78, 85),<br>daunorubicin 30 mg/m2 (days 8, 15, 22, 29),<br><br>E. coli, l-asparaginase 5,000 IU/m2/d (days 12, 15, 18, 21, 24, 27,<br>30, 33, 50, 52, 54, 57, 59, 61, 78, 80, 82, 85, 87, 89),<br>cyclophosphamide 1,000 mg/m2/d (days 36, 64),<br>6-mercaptopurine 60 mg/m2/d (days 36-49, 64-77),<br>cytarabine 75 mg/m2/d (days 37-40, 43-46, 65-68, 72-75),<br>intrathecal methotrexate, age-adjusted dosage, max. 12 mg/d (days 1, 12, 33, 37, 44, 51, 58, if CNS positive or traumatic LP additional doses on days 18, 27)
**consolidation therapy**	Protocol M<br>6-mercaptopurine 25 mg/m2/d (days 1-56),<br>methotrexate 2,000 mg/m2 (days 8, 22, 36, 50) – SRG ALL-BCP or 5,000 mg/m2 (days 8, 22, 36, 50) – SRG ALL-T<br>intrathecal methotrexate, age-adjusted dosage, max. 12 mg/d (days 8, 22, 36, 50)	Protocol M<br>6-mercaptopurine 25 mg/m2/d (days 1-56),<br>methotrexate 5,000 mg/m2/d (days 8, 22, 36, 50),<br>intrathecal methotrexate, age-adjusted dosage, max. 12 mg/d (days 8, 22, 36, 50)	Protocol HR1, HR2, HR3<br>dexamethasone 20 mg/m2/d (days 1-5 prot. HR1, HR2, HR3)<br>vincristine 1,5 mg/m2/d, max. 2 mg (days 1, 6 prot. HR1),<br>methotrexate 5,000 mg/m2/d (day 1 prot. HR1, HR2),<br>cyclophosphamide 200 mg/m2/d (5 doses on days 2, 3, 4 prot. HR1),<br>cytarabine 2,000 mg/m2/d (2 doses on day 5 prot. HR1, 4 doses on days 1 and 2 prot. HR3),<br>l-asparaginase 25,000 IU/m2/d (day 6 prot. HR1, HR2, HR3),<br>vindesine 3 mg/m2/d, max. 5 mg (days 1, 6 prot. HR2),<br>ifosfamide 800 mg/m2/d (5 doses on days 2, 3, 4 prot. HR2),<br>daunorubicin 30 mg/m2/d (day 5 prot. HR2),<br>etoposide 100 mg/m2/d (5 doses on days 3, 4, 5 prot. HR3),

			intrathecal methotrexate+cytarabine+prednizolone, age-adjusted dosage, max. 12+30+10 mg/d (day 1 prot. HR1; day 1 and 5 prot. HR2, day 5 prot. HR3)<br>HR1′, HR2′, HR3′ as in HR
**reinduction therapy**	Protocol II<br>dexamethasone 10 mg/m2/d (days 1-21), vincristine 1.5 mg/m2/d, max. 2 mg (days 8, 15, 22, 29),<br>doxorubicin 30 g/m2/d (days 8, 15, 22, 29),<br>E. coli L-asparaginase 10,000 IU/m2/d (days 8, 11, 15, 18),<br>cyclophosphamide 1,000 mg/m2/d (day 36),<br>6-thioguanine 60 mg/m2/d (days 36-49), cytarabine 75 mg/m2/d (days 38-41, 45-48),<br>intrathecal methotrexate, age-adjusted dosage, max. 12 mg/d (days 38, 45), if CNS positive or traumatic LP additional doses on days 1, 18)	Protocol II<br>dexamethasone 10 mg/m2/d (days 1-21), vincristine 1.5 mg/m2/d, max. 2 mg (days 8, 15, 22, 29),<br>doxorubicin 30 g/m2/d (days 8, 15, 22, 29),<br>E. coli L-asparaginase 10,000 IU/m2/d (days 8, 11, 15, 18),<br>cyclophosphamide 1,000 mg/m2/d (day 36),<br>6-thioguanine 60 mg/m2/d (days 36-49), cytarabine 75 mg/m2/d (days 38-41, 45-48),<br>intrathecal methotrexate, age-adjusted dosage, max. 12 mg/d (days 38, 45), if CNS positive or traumatic LP additional doses on days 1, 18)	Protocol II<br>dexamethasone 10 mg/m2/d (days 1-21), vincristine 1.5 mg/m2/d, max. 2 mg (days 8, 15, 22, 29),<br>doxorubicin 30 mg/m2/d (days 8, 15, 22, 29),<br>E. coli L-asparaginase 10,000 IU/m2/d (days 8, 11, 15, 18),<br>cyclophosphamide 1,000 mg/m2/d (day 36),<br>6-thioguanine 60 mg/m2/d (days 36-49), cytarabine 75 mg/m2/d (days 38-41, 45-48),<br>intrathecal methotrexate, age-adjusted dosage, max. 12 mg/d (days 38, 45), if CNS positive or traumatic LP additional doses on days 1, 18
maintenance therapy	methotrexate 20 mg/m2 (once per week),<br>6-mercaptopurine 50 mg/m2/d (once per day),<br>intrathecal methotrexate, age-adjusted dosage, max. 12 mg/d (every 4 weeks, 2-4 doses in selected groups of patients); no MTX i.th. if CRT indicated	methotrexate 20 mg/m2 (once per week),<br>6-mercaptopurine 50 mg/m2/d (once per day),<br>intrathecal methotrexate, age-adjusted dosage, max. 12 mg/d (every 4 weeks, 2-4 doses in selected groups of patients) ; no MTX i.th. if CRT indicated	methotrexate 20 mg/m2 (once per week),<br>6-mercaptopurine 50 mg/m2/d (once per day),<br>therapeutic or prophylactic CRT
Cumulative asparaginase dose	80 000 u/m2	80 000 u/m2	290 000 u/m2
